# Unilateral biportal endoscopic decompression compared with tubular or uniportal endoscopic decompression for lumbar spinal stenosis: a systematic review and meta-analysis

**DOI:** 10.3389/fsurg.2026.1805531

**Published:** 2026-04-20

**Authors:** Jie Song, Jun Li, Xiu-Lei Xu, Quan Sun

**Affiliations:** 1 Department of Orthopedics, Sir Run Run Shaw Hospital, Alaer Hospital, Zhejiang University School of Medicine, Urumqi, Urumqi, Xinjiang; 2 Department of Orthopedic, Xinjiang Production and Construction Corps General Hospital, Urumqi, Urumqi, Xinjiang

**Keywords:** lumbar spinal stenosis, meta-analysis, tubular decompression, unilateral biportal endoscopic decompression, uniportal endoscopic decompression

## Abstract

**Background:**

Comparative evidence regarding unilateral biportal endoscopic decompression vs. tubular decompression or uniportal endoscopic decompression for lumbar spinal stenosis remains limited and inconsistent.

**Methods:**

We conducted a systematic review and meta-analysis of comparative clinical studies that evaluated UBE vs. tubular decompression or uniportal endoscopic decompression for lumbar spinal stenosis. Pooled analyses were performed for predefined outcomes, and comparator-specific analyses were additionally performed to improve interpretability.

**Results:**

A total of 1,395 patients from 10 studies were included in this meta-analysis. Overall complications and dural tear or cerebrospinal fluid leak events were lower in the unilateral biportal endoscopic decompression group than in the pooled control group. The unilateral biportal endoscopic decompression group also showed modestly lower ODI and pain scores at the final follow-up, although the magnitude of benefit varied across outcomes. In addition, the changes in the dural sac cross-sectional area after surgery also favored unilateral biportal endoscopic decompression, with low to moderate heterogeneity.

**Conclusions:**

For lumbar spinal stenosis, UBE may provide comparable overall safety and modest advantages in selected perioperative, clinical, and radiological outcomes compared with tubular or uniportal endoscopic decompression. However, the clinical relevance of some statistically significant differences remains uncertain, and further high-quality comparative studies are required.

## 1 Introduction

Degenerative lumbar spinal stenosis (LSS) is a common cause of leg pain, neurogenic claudication, and functional impairment in elderly individuals, and is also the leading indication for spinal surgical intervention worldwide (1, 2). Recent systematic reviews have highlighted that while LSS is often treated non-operatively, a significant subgroup of patients ultimately requires surgery because symptoms remain debilitating despite conservative care (3).

When surgery is selected, decompression is widely used to address symptomatic neural element compression (4). Longer-term follow-up from comparative cohorts has shown that surgical and non-operative strategies can both yield improvement over time, while between-group differences may persist for selected outcomes in certain settings (5). Randomized evidence has also reported improvements in both arms, with an average advantage for surgery in disability and pain outcomes at short- to midterm follow-up (6). Simultaneously, evidence synthesis has reported heterogeneity among included trials and care pathways, which still fuels debates on the best criteria for patient selection and technique (7).

Traditional open decompression can be effective; however, there is anxiety among patients and surgeons that this approach may cause greater disruption of the paraspinal soft tissues, leading to more postoperative pain and delayed recovery. Such concerns have contributed to the rapid adoption of minimally invasive decompression techniques (8). Among the minimally invasive options, uniportal endoscopic decompression and tubular microscopic decompression aim to reduce tissue trauma while maintaining adequate canal decompression (9). Prospective comparative data have shown that uniportal endoscopic decompression can achieve clinical improvement with perioperative profiles that may differ from those of tubular techniques (10).

Unilateral biportal endoscopic decompression (UBE) has emerged as an alternative endoscopic strategy that separates visualization and instrumentation into two portals, which may expand maneuverability and the operative field compared with uniportal configurations (11). Clinical series and methodological reports have described the feasibility of biportal decompression for degenerative lumbar canal stenosis and outlined technique-specific considerations, including irrigation management and instrument freedom (12). Comparative clinical studies have also evaluated biportal endoscopic surgery against conventional approaches for LSS, reporting postoperative improvements in pain and function, with differences in perioperative parameters across techniques (13). Two-year follow-up investigations have further reported clinical and radiological outcomes after UBE using arthroscopy-assisted visualization (14). Technical modifications, such as contralateral procedures, have been developed to treat asymmetric stenosis while achieving decompression and preserving the facet (15).

UBE has recently emerged as an alternative endoscopic approach. First, it could potentially minimize muscle trauma while still achieving sufficient neural decompression. Meanwhile, UBE has been compared with tubular and uniportal endoscopic techniques in clinical studies, despite the majority of the studies being single-center ones with a small sample size, mixed indications, and outcome measures. Previous systematic reviews have also compared UBE with open or microscopic decompression; however, direct comparisons among endoscopic methods for degenerative LSS are lacking. Consequently, it remains unclear how UBE compares with other minimally invasive endoscopic techniques in terms of complication profiles, perioperative metrics, patient-reported outcomes, and radiological decompression. Because tubular decompression and uniportal endoscopic decompression represent distinct minimally invasive strategies with different technical features, learning curves, and visualization characteristics, a direct comparison with UBE warrants careful interpretation. Thus, we performed a systematic review and meta-analysis of comparative studies to (1) compare UBE with tubular decompression and uniportal endoscopic decompression for degenerative LSS, (2) assess safety, perioperative, functional, pain, and radiological outcomes, and (3) explore whether the pooled findings remained consistent across different comparator types.

## 2 Materials and methods

This meta-analysis and systematic review evaluated UBE, tubular decompression, and uniportal endoscopic decompression for LSS. The review was written and reported following the Preferred Reporting Items for Systematic Reviews and Meta-Analyses (PRISMA) 2020 statement (16). The PRISMA 2020 explanation and elaboration were consulted for enhancing the coherence of the reporting decisions, if necessary (17). However, this review was not prospectively registered in PROSPERO or any other public registry.

### 2.1 Eligibility criteria

Studies were eligible if they enrolled adults with a diagnosis of LSS and compared UBE decompression with tubular or uniportal endoscopic decompression. In addition, clinical studies such as randomized controlled trials (RCTs) and observational comparative studies were considered eligible. However, case reports, single-arm studies, conference abstracts without adequate outcome data, biomechanical studies, cadaveric studies, and non-comparative designs were excluded. Studies were also excluded if they were focused on tumors, infection, fracture, deformity, or other non-degenerative etiologies. The eligible comparators included tubular microscopic and uniportal endoscopic decompression. Because these comparators differ in their technical characteristics, comparator-specific subgroup analyses were planned where data permitted.

### 2.2 Information sources and search strategy

A systematic search of PubMed, Embase, Web of Science, and CNKI was conducted from database inception to 15 January 2026. The detailed search strategy combined controlled vocabulary and free-text terms related to lumbar spinal stenosis, unilateral biportal endoscopy, biportal endoscopy, tubular decompression, and uniportal endoscopic decompression. The reference lists of eligible studies and relevant reviews were screened manually.

### 2.3 Study selection

The study selection process was summarized using a PRISMA 2020 flow diagram (16). Based on the PRISMA guidelines, two reviewers independently screened titles and abstracts to identify potentially eligible studies. Then, full-text articles were sought to assess eligibility based on the inclusion criteria. Disagreements were resolved through discussion and, when necessary, through consultation with a third reviewer.

### 2.4 Outcomes

The outcomes included safety, perioperative, patient-reported, and radiological outcomes. Safety outcomes included the overall complications and dural tears or cerebrospinal fluid (CSF) leaks. Perioperative outcomes included estimated blood loss. Patient-reported outcomes included the Oswestry disability index (ODI) and visual analog scale (VAS) scores for leg and back pain at the final follow-up. The radiological outcomes also included postoperative changes in the dural sac cross-sectional area (DSCSA). For the radiological outcomes, greater postoperative enlargement of the dural sac cross-sectional area was considered favorable. Because studies reported this outcome using different scales or summary formats, standardized mean differences were used where appropriate; negative values favored UBE when they reflected a greater postoperative increase in the DSCSA.

### 2.5 Data extraction and data handling

Two reviewers independently extracted data using a standardized form. The extracted data included study design, country, diagnosis, decompression levels, comparator types, sample size, participant characteristics, follow-up duration, and outcome data with corresponding dispersion statistics. For postoperative outcomes, the longest available follow-up duration reported in each study was used as the final follow-up time point.

### 2.6 Risk of bias assessment

**Figure 1:**
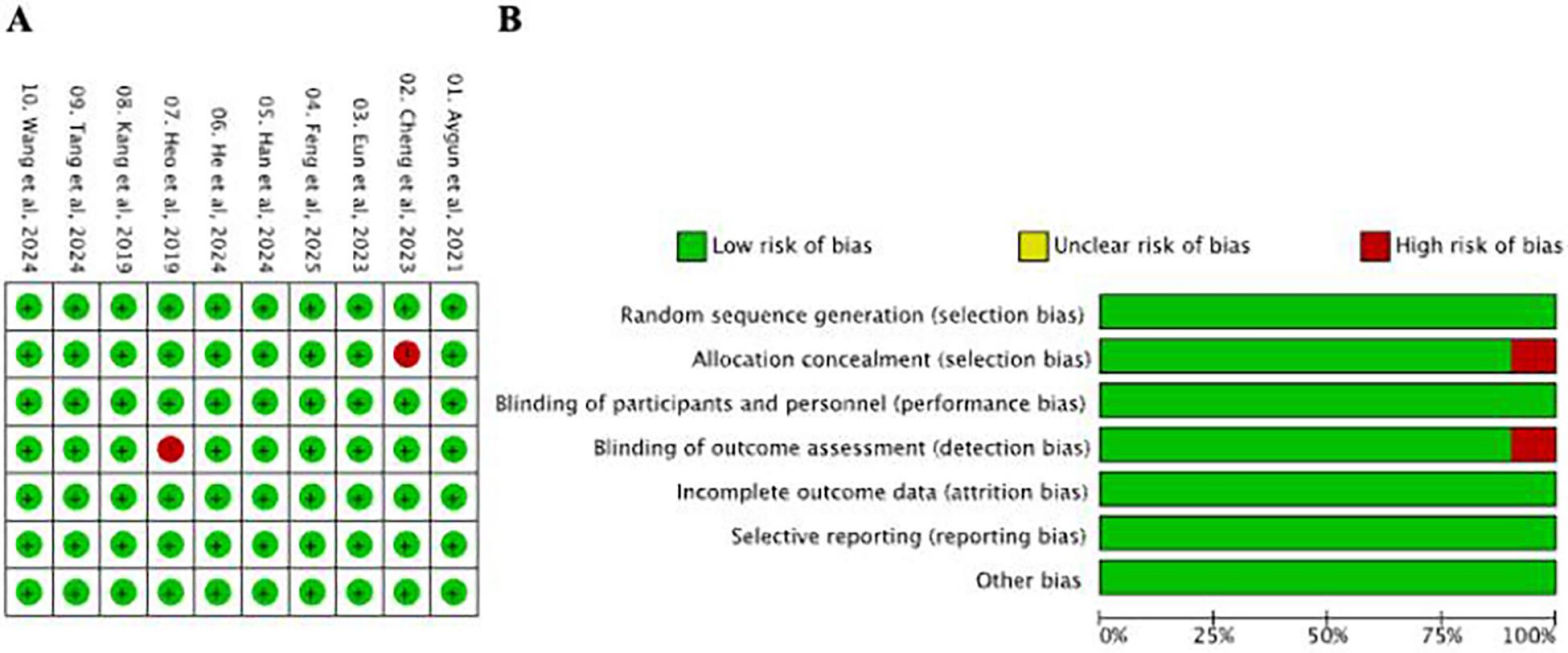
**(A)** Risk of bias summary, and **(B)** risk of bias graph for the included studies. Randomized controlled trials were assessed using the Cochrane risk of bias framework, whereas observational comparative studies were evaluated descriptively with respect to their key methodological limitations.

The risk of bias was assessed independently by two reviewers. For randomized controlled trials, methodological quality was evaluated using the Cochrane risk of bias framework. For comparative observational studies, methodological limitations were descriptively reviewed based on key domains such as selection methods, baseline comparability, outcome assessment, and completeness of follow-up. Disagreements were resolved through discussion and consensus, and the overall methodological profile of the included clinical studies is summarized in Figure 1.

### 2.7 Statistical analysis

All meta-analyses and plot generation were performed using the software RevMan (Version 5.4, Copenhagen) by the Cochrane Collaboration, 2020 (18). For dichotomous outcomes, pooled effects were calculated as risk differences with 95% confidence intervals. For continuous outcomes, pooled effects were calculated as mean differences or standardized mean differences with 95% confidence interval.

Given the expected clinical and methodological heterogeneity across study designs, comparator types, and follow-up durations, random-effect models were used as the primary analytical approach.

Comparator-specific and study-design-based exploratory assessments were considered during the interpretation of the pooled findings, particularly to examine whether the overall direction of effect appeared consistent across different comparator types and study designs. Because the number of studies in several strata was limited, these assessments were not intended to support definitive subgroup conclusions.

Statistical heterogeneity was assessed using the *χ*^2^ test and quantified using *I*^2^ statistics. An *I*^2^ value of <25% was considered low, 25%–50% low to moderate, and >50% substantial heterogeneity (19).

### 2.8 Publication bias

Publication bias was explored using funnel plots for outcomes with a sufficient number of studies. Because most of the pooled outcomes included only a limited number of studies, funnel plot interpretation was considered exploratory rather than definitive.

## 3 Results

### 3.1 Study selection and risk of bias assessment

**Figure 2:**
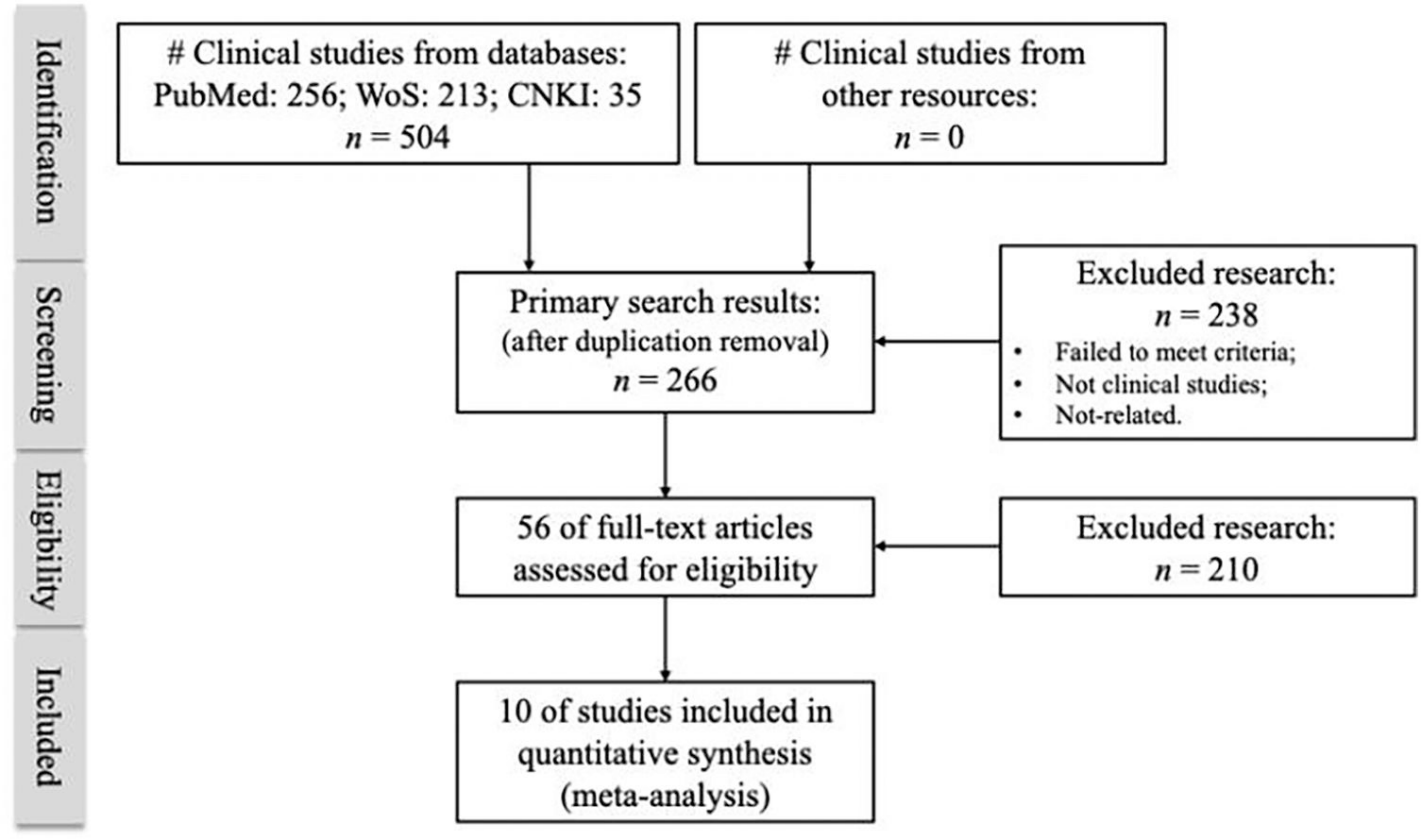
PRISMA flow diagram. A flow diagram of study identification, screening, eligibility assessment, and final inclusion in the systematic review and meta-analysis. Records were identified through database searching and other sources, duplicates were removed, and full texts were assessed against predefined eligibility criteria. The reasons for full-text exclusion are summarized in the diagram. The review was conducted in accordance with the PRISMA 2020 guidelines.

As shown in Table 1 and Figure 2, a total of 1,395 patients were included in the quantitative synthesis, with 706 patients treated with UBE and 689 patients treated with tubular or uniportal endoscopic decompression (20–29).

**Table 1:** Summary of the characteristics of the included comparative studies.

Study	Country	Design	Diagnosis	Levels	Follow-up (months)	Control comparator (s)	Control age (year)	UBE (*n*)	UBE age (year)
01. Aygun et al. (20)	Turkey	Prospective randomized	Degenerative lumbar canal stenosis (single level)	Single level	24	Tubular (*n* = 77)	65.0 ± 9.2	77	64.6 ± 10.1
02. Cheng et al. (21)	China	Retrospective cohort	Lumbar lateral recess stenosis (single level)	Single level	12	Uniportal (*n* = 53)	69.1 ± 7.2	52	69.5 ± 7.3
03. Eun et al. (22)	Korea	Retrospective cohort	Degenerative lumbar spinal stenosis	2–3 levels	12	Tubular (*n* = 48)	68.7 ± 8.7	47	68.2 ± 11.0
04. Feng et al. (23)	China	Retrospective cohort	Degenerative lumbar spinal stenosis	NA	24	Tubular (*n* = 61)	63.4 ± 6.7	70	62.4 ± 9.1
05. Han et al. (24)	China	Retrospective cohort	Lumbar spinal stenosis	Single level	6	Uniportal (*n* = 54)	52.5 ± 19.2	56	58.2 ± 21.0
06. He et al. (24)	China	Retrospective cohort	Degenerative lumbar spinal stenosis (ULBD)	NA	12	Uniportal (*n* = 41)	62.5 ± 8.4	41	67.7 ± 9.0
07. Heo et al. (26)	Korea	Retrospective cohort	Degenerative lumbar spinal stenosis	Single level	12	Tubular (*n* = 33)	63.4 ± 11.1	37	66.7 ± 9.4
08. Kang et al. (27)	Korea	Randomized controlled trial	Degenerative lumbar spinal stenosis	Single level	6	Tubular (*n* = 30)	65.1 ± 8.6	30	67.2 ± 9.5
09. Tang et al. (28)	China	Retrospective cohort	Lumbar spinal stenosis (single level)	Single level	12	Uniportal (*n* = 52)	61.5 ± 7.1	61	65.3 ± 6.9
10. Wang et al. (29)	China	Retrospective cohort	Lumbar spinal stenosis	≤2 levels	6	Uniportal (*n* = 240)	56.7 ± 7.8	235	57.9 ± 6.9

ULBD, unilateral laminotomy for bilateral decompression.

**Figure 3:**
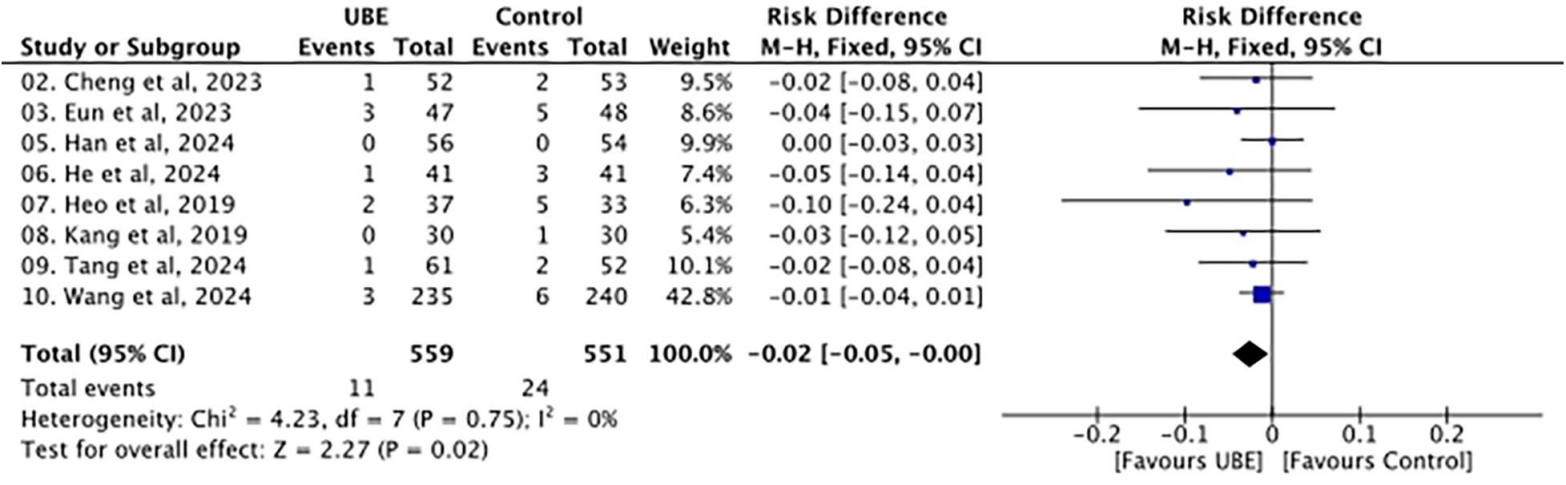
Forest plot of overall complications.

The methodological assessment of the included clinical studies is presented in Figure 1. Overall, the included evidence base showed no obvious concentration of serious methodological concerns in a single aspect, although observational comparative studies should still be interpreted cautiously because of their inherent susceptibility to selection bias and residual confounding. The follow-up duration ranged from 6 to 24 months across studies, with a weighted mean follow-up of approximately 11.7 months.

### 3.2 Overall complications

Overall, complications occurred in 11 of 706 patients in the UBE group and 24 of 689 patients in the pooled control group, corresponding to crude event rates of approximately 1.6% and 3.5%, respectively. As shown in Figure 3, the pooled analysis showed a lower complication rate in the UBE group, with an effect estimate of −0.02 [95% confidence interval (CI) −0.05 to −0.00]. No heterogeneity was found among the studies (*I*^2^ = 0%), and the overall effect was statistically significant.

### 3.3 Dural tear and CSF leak

**Figure 4:**
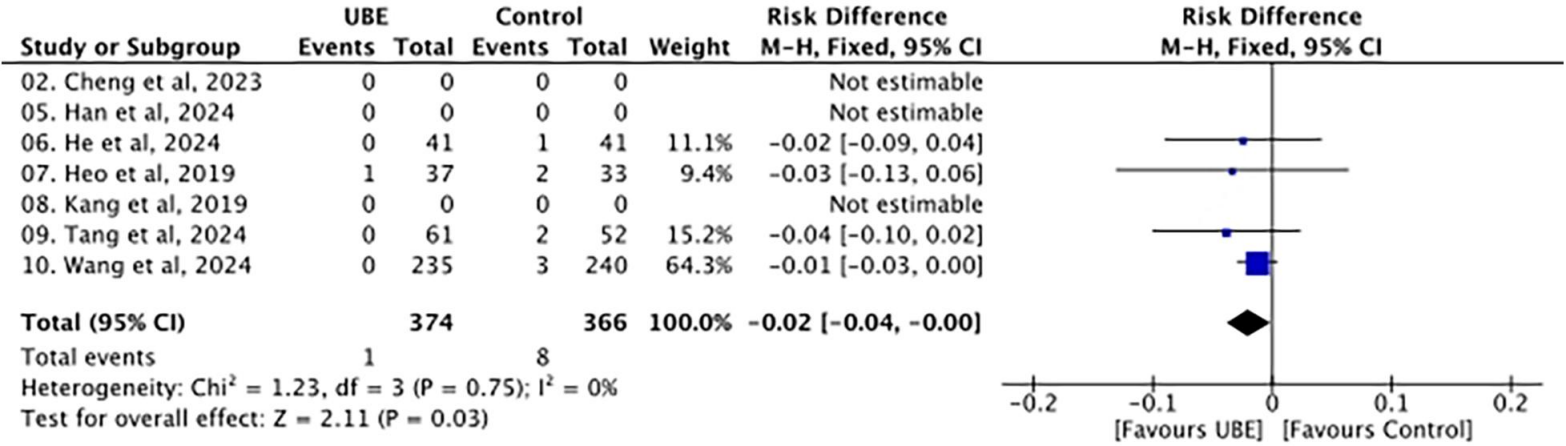
Forest plot of the dural tear or cerebrospinal fluid leak.

Dural tear or CSF leak events were rare, occurring in 1 of 706 patients in the UBE group and 8 of 689 patients in the pooled control group. The pooled effect estimate was −0.02 (95% CI −0.04 to −0.00), indicating fewer events in the UBE group (Figure 4). No heterogeneity was observed between the studies (*I*^2^ = 0%). The pooled effect was statistically significant.

### 3.4 Estimated blood loss

**Figure 5:**
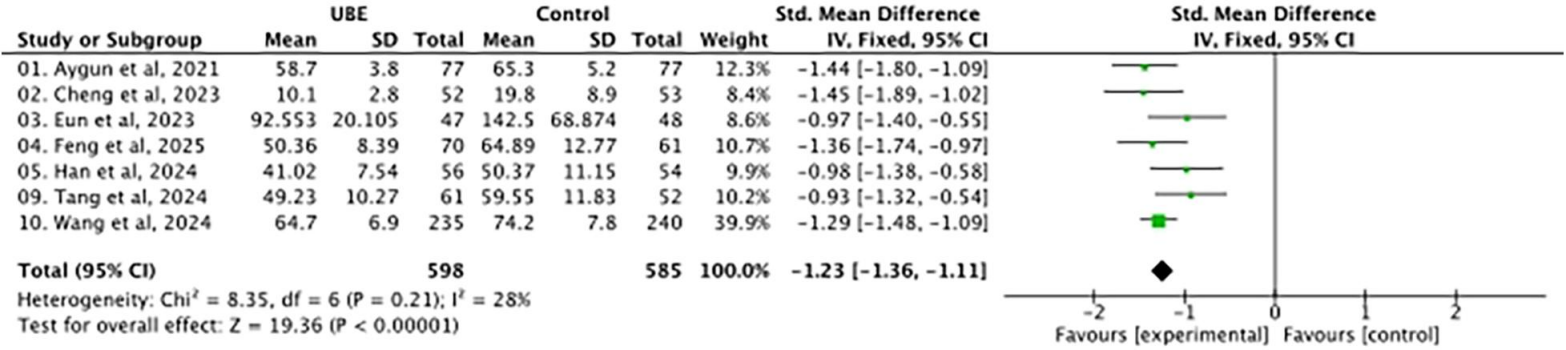
Forest plot of estimated blood loss.

Blood loss was evaluated in 944 patients. Because the estimated blood loss was reported with substantial variation in dispersion and measurement characteristics across studies, standardized mean differences were used. As shown in Figure 5, UBE had less estimated blood loss (EBL) than control surgeries [standardized mean difference (SMD), −0.50, 95% CI −0.86 to −0.13]. Moderate heterogeneity was observed across studies, with an *I*^2^ of 42%, while the combined effect was significant.

### 3.5 Functional outcomes measured by the ODI

**Figure 6:**
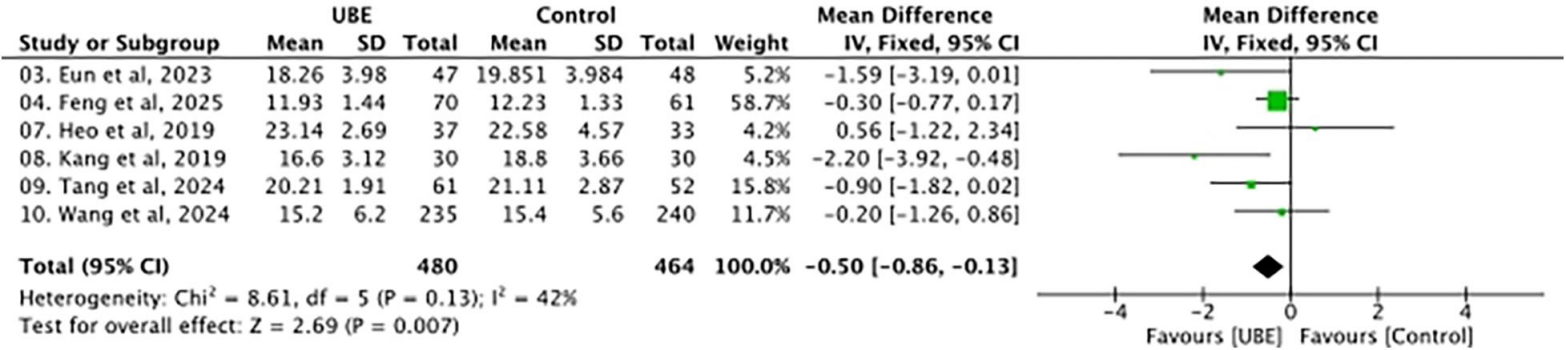
Forest plot of the ODI at the final follow-up.

Final follow-up functional outcomes were assessed in 874 patients using the ODI. A lower ODI was found in the UBE group (SMD −0.34, 95% CI −0.42 to −0.26), as illustrated in Figure 6. The heterogeneity was low to moderate d (*I*^2^=34%), and the pooled result was statistically significant but modest.

### 3.6 Leg pain assessed by the VAS

**Figure 7:**
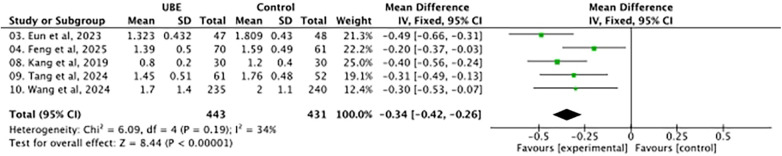
Forest plot of VAS leg pain at the final follow-up.

Leg pain intensity at the final follow-up was reported in 884 patients. Although the pooled VAS leg pain score was statistically lower in the UBE group (MD −0.13, 95% CI −0.22 to −0.04), the absolute difference was small, as shown in Figure 7. Moderate heterogeneity was detected between the studies (*I*^2^ = 48%), and the pooled effect was significant.

### 3.7 Back pain assessed by the VAS

**Figure 8:**
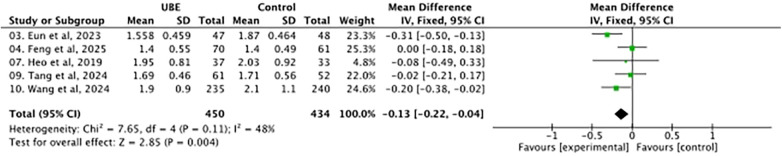
Forest plot of VAS back pain at the final follow-up.

Back pain outcomes were assessed for 1,183 patients. The pooled results showed lower postoperative back pain scores in the UBE group (MD −1.23, 95% CI −1.36 to −1.11). Between-study heterogeneity was moderate (*I*^2^ = 48%). As shown in Figure 8, although the difference was statistically significant, its magnitude was notably larger than that of several other pooled outcomes and should therefore be interpreted cautiously, particularly in light of baseline comparability, study design heterogeneity, and surgeon-dependent factors.

### 3.8 Change in the dural sac cross-sectional area

**Figure 9:**
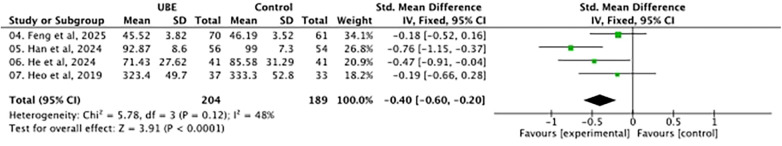
Forest plot of the DSCSA change. A forest plot comparing postoperative changes in the dural sac cross-sectional area between the UBE and the pooled control groups. Standardized mean differences with 95% confidence intervals are presented.

The postoperative changes in the DSCSA were assessed in 393 patients. The pooled analysis favored UBE (SMD −0.40, 95% CI −0.60 to −0.20) with moderate heterogeneity (*I*^2^ = 48%). As shown in Figure 9, negative SMD values indicate a greater postoperative increase in the DSCSA in the UBE group compared with that in the control group.

**Figure 10:**
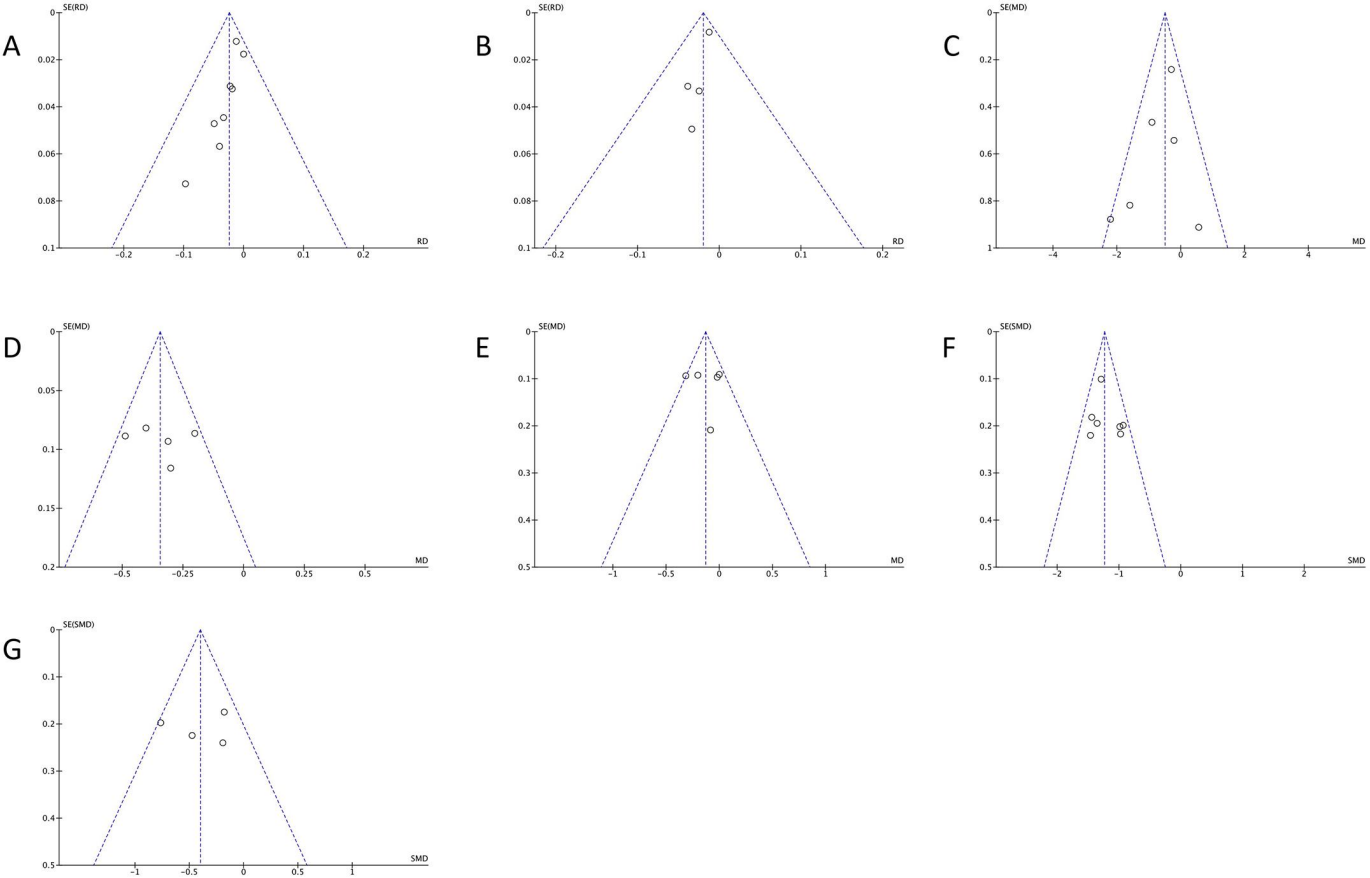
Funnel plots of **(A)** overall complications; **(B)** dural tear and/or cerebrospinal fluid leak; **(C)** ODI at the final follow-up; **(D)** VAS score of the leg; **(E)** VAS score of the back; **(F)** estimated blood loss; and **(G)** DSCSA change.

### 3.9 Publication bias assessment

As shown in Figure 10, visual inspection of the funnel plots did not suggest obvious asymmetry for most outcomes; however, these findings should be interpreted cautiously because the number of studies contributing to each pooled analysis was small, limiting the reliability of the funnel plot assessment.

### 3.10 Subgroup and sensitivity analyses

Comparator-specific and study-design-based exploratory assessments were considered during the interpretation of the pooled findings. Because the number of studies in several outcome-specific strata was limited, these analyses were primarily used to assess the consistency of direction rather than to support definitive subgroup conclusions.

## 4 Discussion

Previous systematic reviews have investigated the clinical outcomes of biportal endoscopic decompression relative to those of uniportal endoscopy and other surgical approaches. A 2024 meta-analysis found a lower ODI at 12 months and larger postoperative dural sac area enlargement after biportal endoscopic decompression than uniportal decompression, with similar safety profiles and complication rates between the methods (30). Another systematic review reported that there were no major differences between biportal and uniportal endoscopic decompression techniques in most outcomes, although the biportal technique did seem to provide better ODI scores at the final evaluation (31). These results are consistent with the overall observations in the current analysis.

UBE has also been described in case series and other non-comparative clinical reports. A 2024 systematic review of biportal endoscopic decompression found significant improvements in VAS back, VAS leg, and ODI scores from pre-to postoperative follow-up in LSS (32). Cohort investigations have also demonstrated clinical amelioration and enlargement of the spinal canal after UBE-unilateral laminotomy for bilateral decompression (ULBD) in individuals with severe LSS (33). These studies contextualize the combined clinical patterns reported in this research.

Comparative clinical research has explored the application of unilateral biportal endoscopic techniques in different operative settings. Wu et al. compared uniportal and biportal decompression and reported similar operative duration, blood loss, and length of hospital stay between techniques, with comparable outcomes at multiple follow-up intervals (34). In addition, another systematic cohort analysis by Ouyang et al. indicated that unilateral biportal endoscopy is effective in treating single-segment degenerative lumbar stenosis and represents an alternative to traditional open decompression surgery (35).

Radiological comparisons between unilateral biportal endoscopy and other decompression methods have also been reported. According to a study by Ross et al., tubular decompression and UBE demonstrated similar clinical outcomes, with differences in radiological decompression measures noted between the techniques (36). A 2024 analysis of ULBD with UBE versus uniportal endoscopic ULBD described larger DSCSA expansion and contralateral facet resection angles with biportal methods (37), further contextualizing radiological trends in the extent of decompression.

Evidence synthesis of complications associated with unilateral biportal endoscopic spine surgery has been reported in recent literature. Meta-analyses have quantified overall complication rates and specific adverse events following UBE procedures, describing dural tears and neurological symptoms among the reported complications (38). Literature reviews on complication profiles pertaining to endoscopic decompression techniques have also been conducted to characterize the safety aspects of these techniques (39).

Investigations into technique-specific factors such as learning curves and operative experience have been undertaken. A 2024 meta-analysis of learning curve data on reported clinical and perioperative profiles of unilateral biportal endoscopic spine surgery may be related to some outcome heterogeneity across studies (40). These analyses underscore the procedural factors that may influence outcomes.

An important methodological consideration is that tubular decompression and uniportal endoscopic decompression are distinct minimally invasive techniques rather than interchangeable techniques. They differ in terms of visualization, instrumentation, working corridor, and learning curve, and these differences may influence perioperative profiles and patient-reported outcomes. Pooling them into a single comparator group provided an overall summary but may have also introduced clinical heterogeneity and obscured technique-specific effects. Therefore, the present findings should be interpreted together with comparator-specific analyses, where available, rather than as evidence that UBE is uniformly superior across all minimally invasive decompression strategies.

To date, the UBE technique is supported only by a small number of comparable and non-comparable studies with varying outcome definitions and follow-up durations. Well-designed prospective randomized controlled studies with uniform outcome definitions and longer follow-up are still required to disclose the comparative efficacy of endoscopic techniques more definitively. Additional investigations with uniform reporting of patient-oriented outcomes, imaging-based decompression measures, and cost analyses are likely to add further knowledge to aid surgical decision-making. More broadly, statistical significance should not be equated with clinical importance. In this study, for several pooled endpoints, particularly leg pain, the absolute differences were modest, and their practical relevance may be limited, even if the pooled effect reaches nominal statistical significance.

Considering the practical aspects, the current findings support UBE as a reasonable minimally invasive alternative rather than a clearly superior technique. Its potential advantages may be more relevant in selected operative settings; however, the clinical importance of some pooled differences, particularly for patient-reported pain outcomes, remains uncertain. Future research should focus on high-quality randomized trials with standardized outcome reporting, longer-term follow-up, and inclusion of patient-reported quality-of-life and cost-effectiveness assessments. Such investigations will be vital to optimize patient selection, characterize the learning curve, and more narrowly define the role of UBE in the evolving minimally invasive surgical treatment options for degenerative LSS.

## 5 Conclusion

In this systematic review and meta-analysis, UBE exhibited comparable overall safety and possible advantages in selected perioperative, functional, pain, and radiological outcomes compared with tubular or uniportal endoscopic decompression for LSS. However, several pooled differences were modest, and the clinical relevance of some statistically significant findings remains unclear. Therefore, UBE may be considered a reasonable minimally invasive alternative rather than a definitively superior technique, and further high-quality comparative studies are needed to clarify its technique-specific benefits.

## Data Availability

The original contributions presented in this study are included in the article/Supplementary Material, further inquiries can be directed to the corresponding author/s.

## References

[1] Katz JN Harris MB. Clinical practice. Lumbar spinal stenosis. N Engl J Med. (2008) 358(8):818–25. 10.1056/NEJMcp0708097 18287604

[2] Lurie J Tomkins-Lane C. Management of lumbar spinal stenosis. Br Med J. (2016) 352:h6234. 10.1136/bmj.h6234 26727925 PMC6887476

[3] Katz JN Zimmerman ZE Mass H Makhni MC. Diagnosis and management of lumbar spinal stenosis: a review. Jama. (2022) 327(17):1688–99. 10.1001/jama.2022.5921 35503342

[4] Weinstein JN Tosteson TD Lurie JD Tosteson ANA Blood E Hanscom B Surgical versus nonsurgical therapy for lumbar spinal stenosis. N Engl J Med. (2008) 358(8):794–810. 10.1056/NEJMoa0707136 18287602 PMC2576513

[5] Weinstein JN Tosteson TD Lurie JD Tosteson A Blood E Herkowitz H Surgical versus nonoperative treatment for lumbar spinal stenosis four-year results of the spine patient outcomes research trial. Spine (Phila Pa 1976). (2010) 35(14):1329–38. 10.1097/BRS.0b013e3181e0f04d 20453723 PMC3392200

[6] Malmivaara A Slätis P Heliövaara M Sainio P Kinnunen H Kankare J Surgical or nonoperative treatment for lumbar spinal stenosis? A randomized controlled trial. Spine (Phila Pa 1976). (2007) 32(1):1–8. 10.1097/01.brs.0000251014.81875.6d 17202885

[7] Zaina F Tomkins-Lane C Carragee E Negrini S. Surgical versus non-surgical treatment for lumbar spinal stenosis. Cochrane Database Syst Rev. (2016) 2016(1):Cd010264. 10.1002/14651858.CD010264.pub2 26824399 PMC6669253

[8] Binder DK Schmidt MH Weinstein PR. Lumbar spinal stenosis. Semin Neurol. (2002) 22(2):157–66. 10.1055/s-2002-36539 12524561

[9] Carrascosa-Granada A Velazquez W Wagner R Mazzei AS Vargas-Jimenez A Jorquera M Comparative study between uniportal full-endoscopic interlaminar and tubular approach in the treatment of lumbar spinal stenosis: a pilot study. Global Spine J. (2020) 10(2 Suppl):70–8. 10.1177/2192568219878419 PMC7263328 32528810

[10] Süner HI Castaño JP Vargas-Jimenez A Wagner R Mazzei AS Velazquez W Comparison of the tubular approach and uniportal interlaminar full-endoscopic approach in the treatment of lumbar spinal stenosis: our 3-year results. World Neurosurg. (2023) 173:e148–55. 10.1016/j.wneu.2023.02.022 36775236

[11] Hwa Eum J Hwa Heo D Son SK Park CK. Percutaneous biportal endoscopic decompression for lumbar spinal stenosis: a technical note and preliminary clinical results. J Neurosurg Spine. (2016) 24(4):602–7. 10.3171/2015.7.Spine15304 26722954

[12] Pao JL Lin SM Chen WC Chang CH. Unilateral biportal endoscopic decompression for degenerative lumbar canal stenosis. J Spine Surg. (2020) 6(2):438–46. 10.21037/jss.2020.03.08 32656381 PMC7340817

[13] Choi DJ Kim JE. Efficacy of biportal endoscopic spine surgery for lumbar spinal stenosis. Clin Orthop Surg. (2019) 11(1):82–8. 10.4055/cios.2019.11.1.82 30838111 PMC6389528

[14] Kim JE Choi DJ. Clinical and radiological outcomes of unilateral biportal endoscopic decompression by 30° arthroscopy in lumbar spinal stenosis: minimum 2-year follow-up. Clin Orthop Surg. (2018) 10(3):328–36. 10.4055/cios.2018.10.3.328 30174809 PMC6107815

[15] Kim JS Park CW Yeung YK Suen TK Jun SG Park JH. Unilateral bi-portal endoscopic decompression via the contralateral approach in asymmetric spinal stenosis: a technical note. Asian Spine J. (2021) 15(5):688–700. 10.31616/asj.2020.0119 33189115 PMC8561150

[16] Page MJ McKenzie JE Bossuyt PM Boutron I Hoffmann TC Mulrow CD The PRISMA 2020 statement: an updated guideline for reporting systematic reviews. Br Med J. (2021) 372:n71. 10.1136/bmj.n71 33782057 PMC8005924

[17] Page MJ Moher D Bossuyt PM Boutron I Hoffmann TC Mulrow CD PRISMA 2020 Explanation and elaboration: updated guidance and exemplars for reporting systematic reviews. Br Med J. (2021) 372:n160. 10.1136/bmj.n160 33781993 PMC8005925

[18] Review Manager 5. Version 5.4. The Cochrane Collaboration. (2020).

[19] Higgins JP Thompson SG Deeks JJ Altman DG. Measuring inconsistency in meta-analyses. Br Med J. (2003) 327(7414):557–60. 10.1136/bmj.327.7414.557 12958120 PMC192859

[20] Aygun H Abdulshafi K. Unilateral biportal endoscopy versus tubular microendoscopy in management of single level degenerative lumbar canal stenosis: a prospective study. Clin Spine Surg. (2021) 34(6):E323–8. 10.1097/bsd.0000000000001122 33470660 PMC8225231

[21] Cheng X Wu Y Chen B Tang J. A comparative study of unilateral biportal endoscopic decompression and percutaneous transforaminal endoscopic decompression for geriatric patients with lumbar lateral recess stenosis. J Pain Res. (2023) 16:2241–49. 10.2147/jpr.S413502 37409162 PMC10319288

[22] Eun D-C Lee Y-H Park J-O Suk KS Kim H Moon SH A comparative analysis of bi-portal endoscopic spine surgery and unilateral laminotomy for bilateral decompression in multilevel lumbar stenosis patients. J Clin Med. (2023) 12(3):1033. 10.3390/jcm12031033 36769686 PMC9918291

[23] Feng X Wang B Ding J Niu B Muhetaer W Yang H Unilateral biportal endoscopic versus microscopic discectomy in degenerative lumbar spinal stenosis: a prospective cohort study. Medicine (Baltimore). (2025) 104(21):1–6. 10.1097/MD.0000000000042594 PMC12113972 40419907

[24] Han G Ma L Cheng Y Zheng K Qi J Dong Z Comparison of effectiveness between unilateral biportal endoscopic and uniportal interlaminar endoscopic decompression in the treatment of lumbar spinal stenosis. Zhongguo Xiu Fu Chong Jian Wai Ke Za Zhi. (2024) 38(3):324–30. 10.7507/1002-1892.202312029 38500426 PMC10982040

[25] He B-l Zhu Z-C Lin L-Q Sun JF Huang YH Meng C Biportal versus uniportal endoscopic technique in unilateral laminectomy for bilateral decompression (ULBD) for lumbar spinal stenosis. Asian J Surg. (2024) 47(1):112–7. 10.1016/j.asjsur.2023.05.068 37331857

[26] Heo DH Lee DC Park CK. Comparative analysis of three types of minimally invasive decompressive surgery for lumbar central stenosis: biportal endoscopy, uniportal endoscopy, and microsurgery. Neurosurg Focus. (2019) 46(5):E9. 10.3171/2019.2.FOCUS197 31042664

[27] Kang T Park SY Kang CH Lee SH Park JH Suh SW. Is biportal technique/endoscopic spinal surgery satisfactory for lumbar spinal stenosis patients?: a prospective randomized comparative study. Medicine (Baltimore). (2019) 98(18):1–7 10.1097/MD.0000000000015451 PMC6504265 31045817

[28] Tang Z Tan J Shen M Yang H. Comparative efficacy of unilateral biportal and percutaneous endoscopic techniques in unilateral laminectomy for bilateral decompression (ULBD) for lumbar spinal stenosis. BMC Musculoskelet Disord. (2024) 25(1):713. 10.1186/s12891-024-07825-z 39237948 PMC11375995

[29] Wang F Wang R Zhang C Song E Li F. Clinical effects of arthroscopic-assisted uni-portal spinal surgery and unilateral bi-portal endoscopy on unilateral laminotomy for bilateral decompression in patients with lumbar spinal stenosis: a retrospective cohort study. J Orthop Surg Res. (2024) 19(1):167. 10.1186/s13018-024-04621-2 38444008 PMC10916320

[30] Lobo K Łajczak P Rajab N Santos C De Oliveira RR Silva YP Uniportal versus biportal endoscopic decompression for the treatment of lumbar spinal stenosis: a systematic review and updated meta-analysis. Global Spine J. (2025) 15(7):3517–30. 10.1177/21925682251339999 40299717 PMC12040859

[31] Kaen A Park MK Son SK. Clinical outcomes of uniportal compared with biportal endoscopic decompression for the treatment of lumbar spinal stenosis: a systematic review and meta-analysis. Eur Spine J. (2023) 32(8):2717–25. 10.1007/s00586-023-07660-1 36991184

[32] Lv S Lv H He Y Xia X. Efficacy of biportal endoscopic decompression for lumbar spinal stenosis: a meta-analysis with single-arm analysis and comparative analysis with microscopic decompression and uniportal endoscopic decompression. Oper Neurosurg. (2024) 27(2):158–73. 10.1227/ons.0000000000001097 38511959

[33] Hu Y Fu H Yang D Xu W. Clinical efficacy and imaging outcomes of unilateral biportal endoscopy with unilateral laminotomy for bilateral decompression in the treatment of severe lumbar spinal stenosis. Front Surg. (2023) 9:1061566. 10.3389/fsurg.2022.1061566 36684266 PMC9852342

[34] Wu PH Chin BZJ Lee P Woon CY Kim HS George R Ambulatory uniportal versus biportal endoscopic unilateral laminotomy with bilateral decompression for lumbar spinal stenosis-cohort study using a prospective registry. Eur Spine J. (2023) 32(8):2726–35. 10.1007/s00586-023-07620-9 36862219

[35] Ouyang JY Yang QY Chen LL Li Q Zheng YH Luo X A comparative analysis of unilateral biportal endoscopic and open laminectomy in multilevel lumbar stenosis. Front Neurol. (2024) 15:1409088. 10.3389/fneur.2024.1409088 39777310 PMC11704811

[36] Ross N Aleman C Dhenin A Vassal M Lonjon G. Tubular versus unilateral biportal endoscopy: mRI analysis after unilateral laminectomy for bilateral decompression in lumbar spinal stenosis. Eur Spine J. (2025) 34(7):2972–80. 10.1007/s00586-025-08953-3 40448852

[37] He BL Zhu ZC Lin LQ Sun JF Huang YH Meng C Comparison of biportal endoscopic technique and uniportal endoscopic technique in unilateral laminectomy for bilateral decomprssion (ULBD) for lumbar spinal stenosis. Asian J Surg. (2024) 47(1):112–7. 10.1016/j.asjsur.2023.05.068 37331857

[38] Chen Z Zhou H Wang X Liu Z Liu W Luo J. Complications of unilateral biportal endoscopic spinal surgery for lumbar spinal stenosis: a meta-analysis and systematic review. World Neurosurg. (2023) 170:e371–9. 10.1016/j.wneu.2022.11.019 36368457

[39] Chen Y Lin W Lei S Zhang X Ma Y Wang D Comparing the efficacy and safety of unilateral biportal endoscopic decompression with percutaneous endoscopic lumbar decompression for lumbar degenerative diseases: a meta-analysis. World Neurosurg. (2024) 187:e383–98. 10.1016/j.wneu.2024.04.093 38657790

[40] Liu S-X Chen R-S Chen C-M He L-R Jhang S-W Lin G-X. Unilateral biportal endoscopic spine surgery: a meta-analysis unveiling the learning curve and clinical benefits. Original research. Front Surg. (2024) 11:1405519. 10.3389/fsurg.2024.1405519 39575448 PMC11578948

